# Supplementary figures and images for: Evolution of phenotypic plasticity leads to tumor heterogeneity with implications for therapy
Source: PLoS Comput Biol. 2024 Aug 9;20(8):e1012003. doi: 10.1371/journal.pcbi.1012003 (PMC11338451; doi:10.1371/journal.pcbi.1012003)

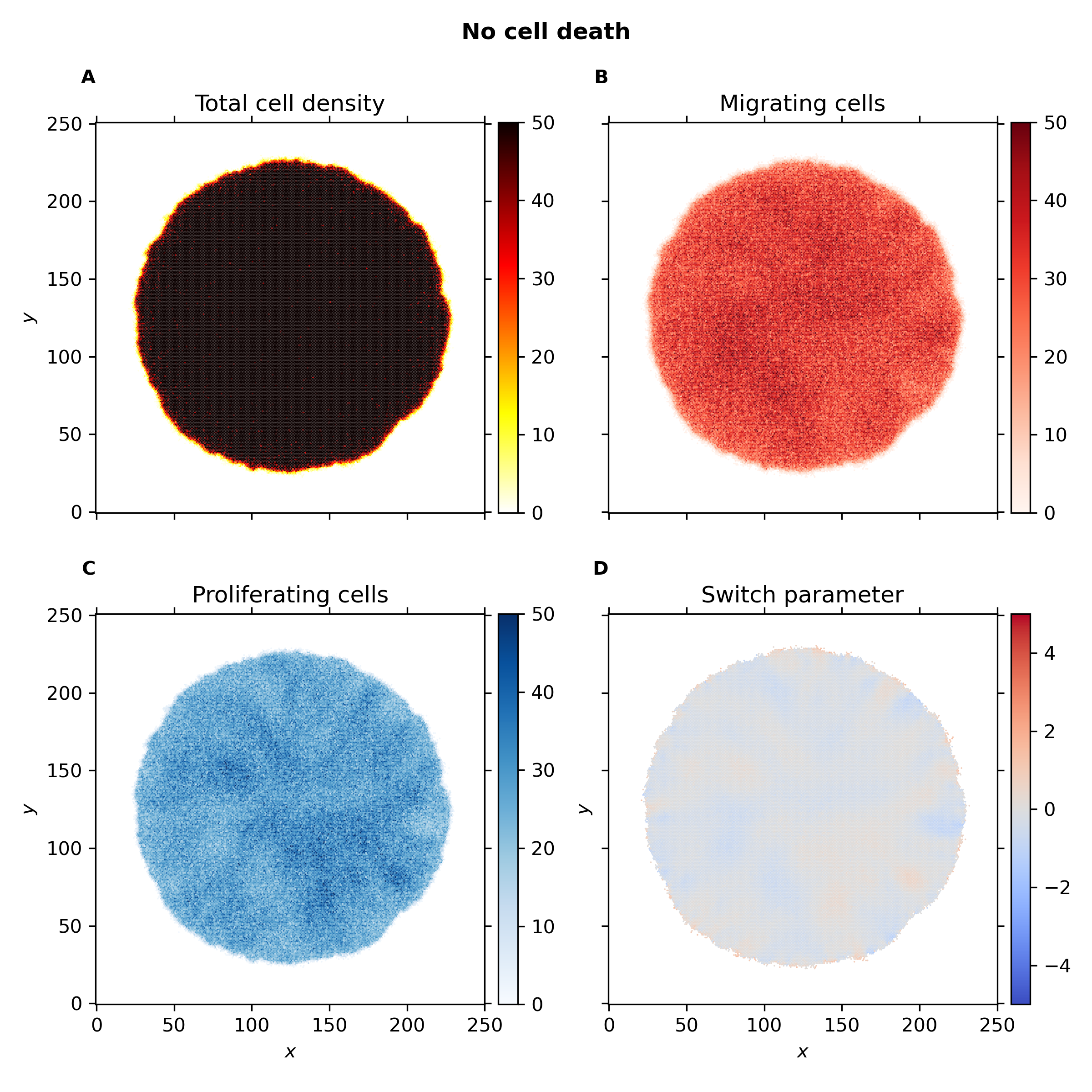

Supplement: S1 Fig — Snapshot of an exemplary simulation on a hexagonal lattice without cell death corresponding to regime 1 in Fig 4. (A) Total cell density, (B) migratory cells, (C) proliferating cells and (D) average local switch parameter κ. Cells with the independent strategy (κ ≈ 0) grow the fastest and dominate the tumor, leading to a mix of migratory and proliferative cells throughout the tumor. Parameters: k = 300, K = 50, L = 250, θ = 0.5, δ = 0. (PNG) [file pcbi.1012003.s002.png]

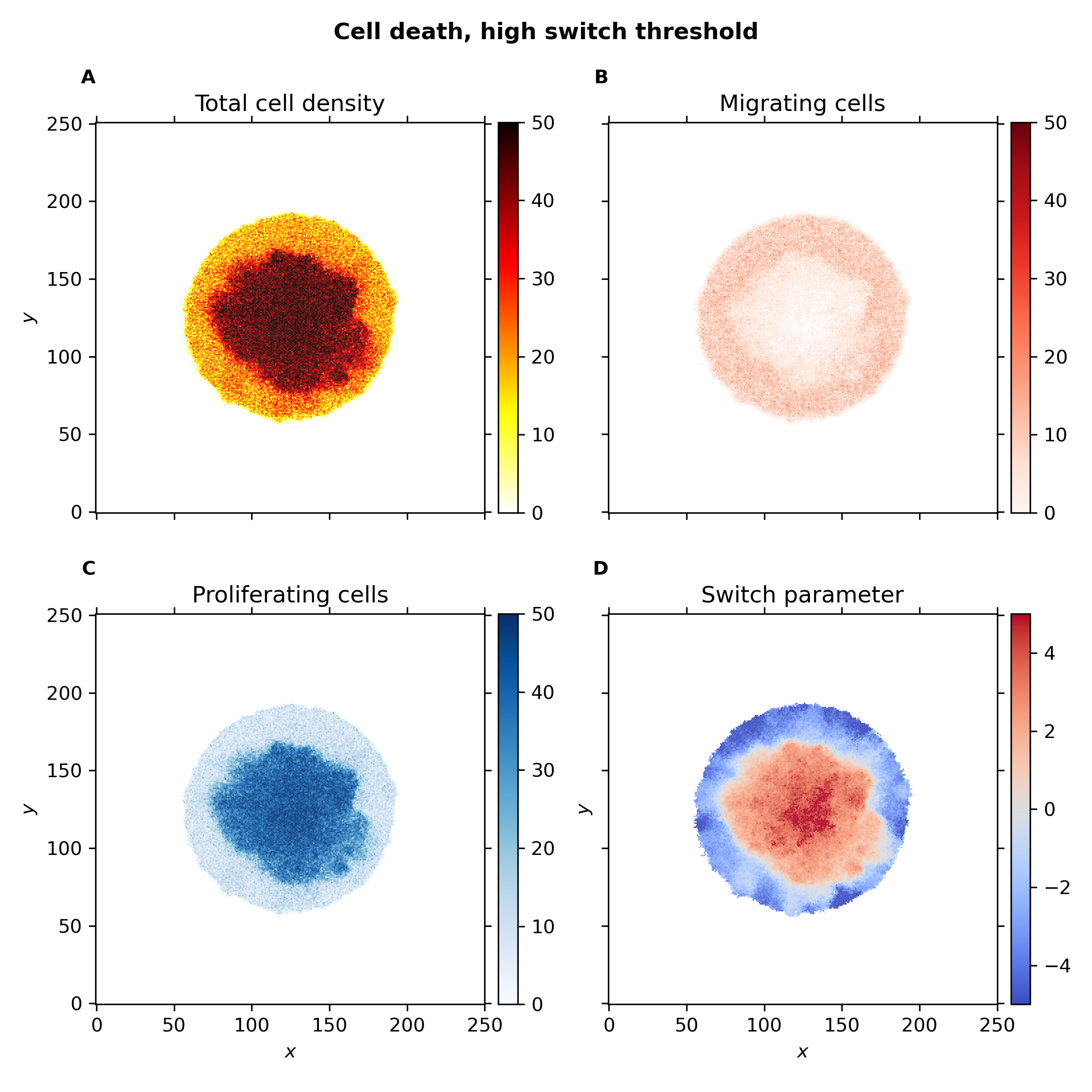

Supplement: S2 Fig — Snapshot of an exemplary simulation on a hexagonal lattice with cell death and low phenotypic switch threshold corresponding to regime 2 in Fig 4. (A) Total cell density, (B) migratory cells, (C) proliferating cells and (D) average local switch parameter κ. Spatial heterogeneity of genotypes (κ values), phenotypes, and density emerges, with a high-density tumor core of proliferating cells with an attractive strategy (κ > 0) surrounded by a low-density tumor rim of migratory cells with a repulsive strategy (κ < 0). Parameters: k = 300, K = 50, L = 250, θ = 0.2, δ = 0.2. (PNG) [file pcbi.1012003.s003.png]

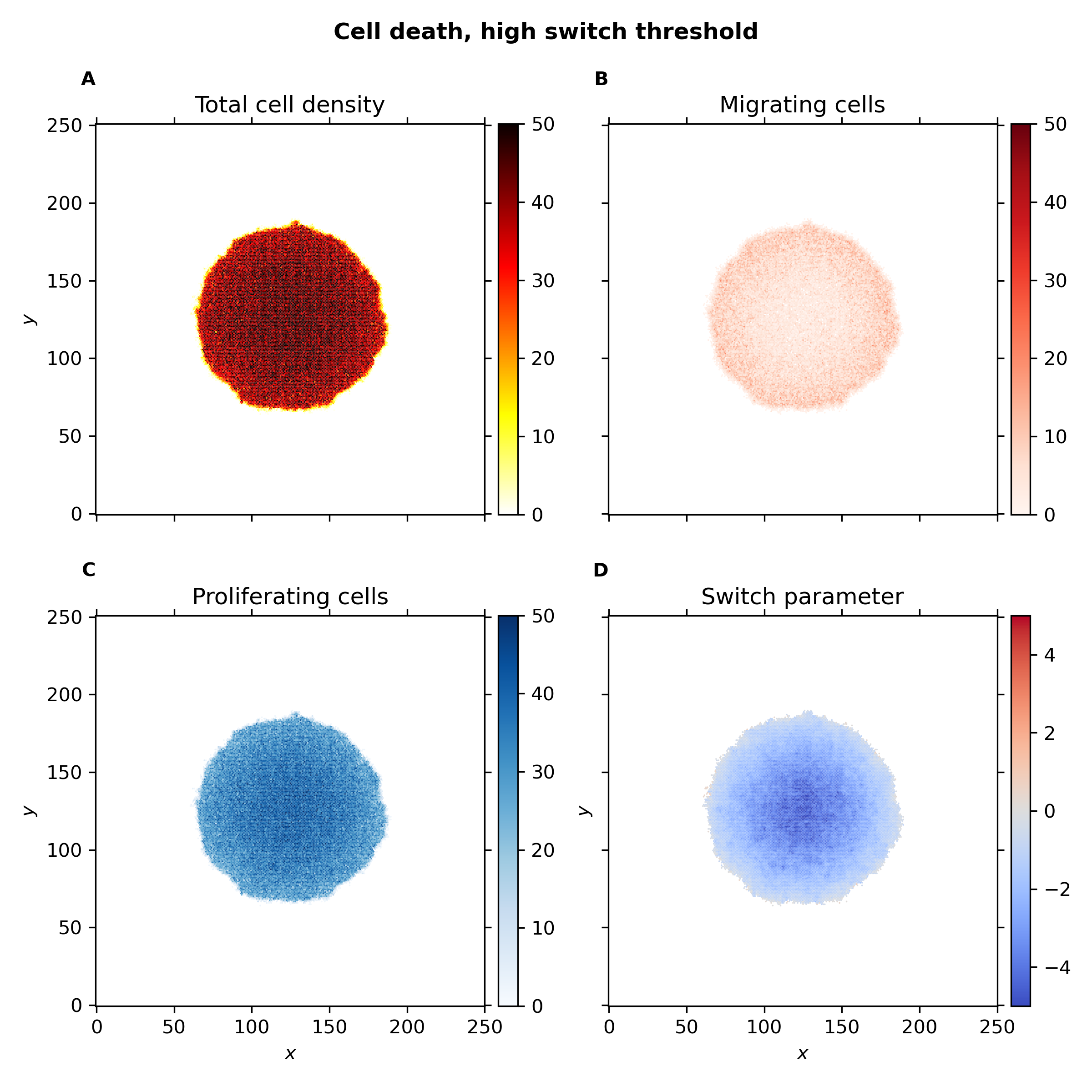

Supplement: S3 Fig — Snapshot of an exemplary simulation on a hexagonal lattice with cell death and low phenotypic switch threshold correponsing to regime 3 in Fig 4. (A) Total cell density, (B) migratory cells, (C) proliferating cells and (D) average local switch parameter κ. The whole tumor evolves towards the repulsive strategy, resulting in a homogeneous tumor of high density with migratory and proliferating cells throughout the tumor. Parameters: k = 300, K = 50, L = 250, θ = 0.9, δ = 0.2. (PNG) [file pcbi.1012003.s004.png]
