# Supplementary material for: Evolution of phenotypic plasticity leads to tumor heterogeneity with implications for therapy
Source: PLoS Comput Biol. 2024 Aug 9;20(8):e1012003. doi: 10.1371/journal.pcbi.1012003 (PMC11338451; doi:10.1371/journal.pcbi.1012003)
Supplement: Attachment — Submitted filename: reviewer_comments_reply.pdf [file pcbi.1012003.s011.pdf]

Evolution of phenotypic plasticity leads to tumor heterogeneity with implications for therapy – Response to Reviewers

We thank the reviewers for their positive evaluation and helpful comments. Please find below a point-by-point response outlining how we incorporated the feedback. The authors' response is *italicized* and changes to the manuscript are additionally indicated by the blue font color. The line numbers refer to the manuscript version with tracked changes.

Reviewer #1

The paper by Syga et al considers a lattice gas cellular automaton model for the phenotypic switching of cancer cells between a proliferative state and a state of propagation. Models of this type are known as go-or-grow models, and they have been used in the modelling of cancer for several years. In this paper the authors focus on the spatial heterogeneity that arises as result of phenotypic and genotypic switches, depending on the microenvironment. The paper is very well written and it shows some interesting results. I recommend publication after my comments below have been addressed.

My main comment relates to the use of kappa, the parameter in the switching probability function. I think its use should be better explained. I was first confused as I realized in the results section that kappa changes over time. In fact, its the main point of the paper. It would be nice to mention this earlier, for example in line 107-109 you could add a comment saying that the changes of kappa during the experiments are the main focus here, or something like that.

We agree with the reviewer that this is the essential point of the paper and should be made explicit earlier. Therefore, we followed the suggestion and added the following sentence:

- *L. 122ff: Due to the variable proliferation behavior caused by the phenotypic switch between migration and proliferation, a constant cell death rate, and a steady influx of variation caused by the mutations, cells are subjected to Darwinian evolution, potentially leading to a complex spatial distribution of κ values.*

Then I tried to understand how kappa changes, and it seems it only changes during a mitosis event, where a daughter cell randomly chooses a kappa "close" to the mother's kappa. This gave me the following understanding. A mother that prefers to move, makes a quick stop to divide, and the daughter cells chose also a kappa that preferentially leads to movement. That's why those kappa values accumulate at the tumor boundary? Is this the right understanding? In other words, from the explanations given I was not sure I understand te selection mechanisms for positive or negative kappa.

Indeed, a cell's kappa value is determined at birth and is similar to the mother cell's value. This is because we associate the kappa value with the genotype and a change as an irreversible genetic change. Therefore, the daughter cell inherits a kappa close to the maternal one. We have further clarified this here:

- *L. 116ff: Mutations. During proliferation, the regulation of the phenotypic switch of the daughter cell d, κ_d, representing the cell's genotype, is drawn from a Gaussian distribution centered on the respective parameter of the mother cell κ_m and has a fixed standard deviation $\Delta\kappa$. This ensures that daughter cells inherit κ values similar to those of the mother cells.*

In the discussion you relate this process to cells decision making. I am not clear what you mean. Based on the above the cells make no decisions whatsoever. Once a kappa is chosen for a cell, it keeps it forever and doesn't make any choice. The choice of go or grow is then given by a fixed probability. No more choice here. The kappa value is given to a cell on its birth, so there is no choice either. I think relating your work to cell decisions is overselling your results, as cell decisions are never modeled here.

We thank the reviewer for this important comment, which we believe must be made crystal clear in the manuscript, as it is the essential point of our paper. In our model, the kappa value alone does not determine the probability of the phenotype. Instead, it determines the phenotypic switch function r_kappa (rho), which also depends on the microenvironment (here: cell density rho). This can be interpreted as follows: each cell processes the available information, i.e., the local cell density, in an individual way (own kappa) to produce a Go or a Grow phenotype. A major change to the previous formulation of the Go or Grow models is that each cell has an individual decision-making mechanism, i.e., individual kappa.

As an example, consider a cell with negative kappa (repulsive go-or-grow strategy, see Fig. 2C): While its kappa value is always fixed, in a low-density environment this cell will prefer to proliferate (grow). However, put in a high-density environment, the cell prefers migration (go). This is why we speak of cell decision-making in this context: The cells regulate their internal variables (phenotype) in response to external input from the microenvironment (cell density) as dictated by their genotype (kappa).

Since we believe that this is an essential point, we have adjusted the text at several positions to stress the argument:

- *L. 60ff: Cells switch between migratory and proliferative phenotypes as dictated by a phenotypic switch function that depends on their individual (fixed) genotype and the (variable) local microenvironment in the form of the local cell density relative to a cell density threshold (switch threshold), see Fig. 1B.*
- *L. 70f: Consequently, the probability of either phenotype is not given by its genotype alone but by the combination of its genotype and microenvironment.*
- *L. 113ff: by a function $r\_\kappa(\rho\_N) \in (0, 1)$ that is determined by the cell's genotype κ and depends on the local microenvironment ρ_N. We define its exact form below.*

Further comments

The model was already published in [23] and some analysis was done there. What is the new contribution here? Please make this more clear.

Indeed, the novelty of this paper over ref. [23] was not fully clear. Therefore, we've added the following clarification to the Methods section:

- *L.125ff: This is the key novelty in this paper compared to [23] where all cells during one simulation followed the same phenotypic switch function $r\_\kappa(\rho)$, \ie, had the same κ value.*

In this paper you do not mention if your model has an Allee effect or not. But the same model in [23] has an Allee effect, hence it should play a role here too? Or not? Please explain.

Yes, the Allee effect is the reason for the tumor extinction after treatment, which we observed in the attractive regime of the evolutionary dynamics. We pointed this out as follows:

- *L.339f: The tumor extinction is a consequence of the strong Allee effect affecting cells with the attractive go-or-grow strategy, as predicted previously [23].*

In equation (2) you only consider monotonic probabilities. Have you thought about non-monotonic functions? Maybe that's a way to model an optimal cell density, that the cells prefer to have.

Indeed, we believe a non-monotonic switch function could result from explicitly considering several microenvironmental inputs to the switch function, like nutrient and oxygen availability, growth factor concentration, etc. We've added a section to the discussion about this. See also our answer to reviewer #2's third comment.

Missing closing bracket in line 267

Thanks for pointing this out, we fixed the missing bracket.

Mean field approximation: While reading this part I was expecting to see a dynamic equation for kappa, like kappa_t = …. Is this possible? Could be interesting.

We did at one point look into this and derived an integro-partial differential equation for rho(kappa, t), i.e., the distribution of kappa values in time. However, the resulting dynamics for kappa were not obvious from this equation. Instead, we quickly realized that simply considering the per-capita growth rate offered a much more straight-forward way to explain the observed dynamics. Therefore, we chose to omit that involved mathematical derivation, which we believe distracts from the story of this paper.

Simulations: Have you done 2-D simulations? It would be nice to see the expanding ring with propagating cells on the outside rim and proliferating cells in the inside. If you have 2-D sims I recommend to add them.

Yes, we confirmed that the dynamics in 2D follows the mean-field analysis and agrees with the 1D simulations. However, we only simulated selected parameter combinations due to the computational cost involved. We created three new supplementary figures and six supplementary movies of the dynamics in the three evolutionary regimes, which we reference throughout the results section. We describe the setup for the 2-D simulations as follows in the method section:

- L. 181ff: We also perform simulations on two-dimensional hexagonal lattices to ensure that our results do not depend on the one-dimensional lattice. We choose a hexagonal lattice over a square lattice to reduce artificial patterns that can occur in spatially discrete models [32]. To this end, we place K = 50 cells in the middle of a hexagonal lattice of sidelength L = 250 and monitor the temporal dynamics for 300 time steps. Other parameters and observables are unchanged compared to the one-dimensional simulations. Due to the computational costs involved, we do not perform a systematic parameter scan but simulate the system for selected parameter sets to ensure that the observed dynamics in two dimensions match those in one dimension qualitatively.

Discussion: Please revise the statements on "cell decision making"

We agree that the way we talk about cell decision-making in the current manuscript can be misleading. We have therefore rephrased the text at several passages and now consistently speak about phenotypic plasticity or phenotypic switch function.

- *L. 439ff: Here, we filled this gap by implicitly modeling the cell decision-making process in the context of the so-called go-or-grow dichotomy using a phenotypic switch function depending on cell density.*
- *L. 451: Experimentally identifying the phenotypic switch function would require studying the respective tumor cell lines in varying microenvironmental contexts.*

Discussion: The discussion is quite repetitive. For example lines 412-416, and 443-447 were already mentioned in the discussion earlier.

We agree that the discussion section was repetitive in some sections. Thus, we have removed the duplicate statements.

Supplement: The supplement is quite useful, so I had a look as well and it is fine.

Reviewer #2

This paper presents a detailed investigation into the evolutionary dynamics of tumor growth, focusing on glioblastoma tumors. The authors employ a mathematical modeling approach to explore the interplay between genetic and phenotypic heterogeneity, and the go-or-grow dichotomy in relation to microenvironmental influences.

The topic of the paper is interesting and addresses a challenging issue in mathematical modeling of cancer and clinical research. The paper is persuasive and clear for the most part. The study is well-structured and considers different scenarios, varying a switching parameter related to different possible tumor microenvironment. Overall, the manuscript presents interesting insights into tumor evolution and its clinical implications. The study incorporates both theoretical modeling with numerical simulations and experimental observations, enhancing the robustness of the findings.

However, some minor revisions should be addressed before publication.

- Although the paper deals with a qualitative rather than a quantitative model, it would benefit from discussing the estimation of model parameters through experimental settings and further validation against clinical data or experimental results. Particularly, it should be discussed whether the values of parameters chosen in Table S2 in the Supplementary Information are model-specific or chosen within a reasonable biological range. If chosen within a biological range, the biological references for these parameters should be added. Moreover, the units of measure should be included in the table.

We agree that the manuscript benefits from a more detailed discussion of the model parameters. Therefore, we expanded Table S2 and Section 3 in the Supplementary Text. In particular, we now explain that the parameters are chosen in such a way that the CA model corresponds to realistic tumor sizes, cell densities, cell cycle times and recurrence times.

- It would be interesting to report the computational cost of 1D simulations and discuss the potential benefits of extending the model to a 2D setting. Commenting on the computational challenges and advantages of transitioning to a higher-dimensional space, as well as discussing how a 2D model could capture spatial complexities and interactions more realistically, would be relevant.

Thank you. Following the reviewers' suggestion, we performed simulations on a two-dimensional hexagonal lattice to confirm the findings made on the one-dimensional lattice and the theoretical predictions. We've added the results in three supplementary figures and six supplementary videos, which we reference throughout the results section. We describe the methodology in a new subsection in Methods as follows:

- *L. 181ff: To ensure that our results do not depend on the one-dimensional lattice, we also perform simulations on two-dimensional hexagonal lattices. We choose a hexagonal lattice over the square lattice to reduce artificial patterns that can occur in spatially discrete models [32]. To this end, we place K = 50 cells in the middle of a hexagonal lattice of sidelength L = 250 and monitor the temporal dynamics for 300 time steps. Other parameters and observables are unchanged compared to the one-dimensional simulations. Due to the computational costs involved, we do not perform a systematic parameter scan but simulate*

the system for selected parameter sets to ensure that the observed dynamics in two dimensions match those in one dimension qualitatively.

- Including perspectives on future research directions would strengthen the conclusion of the paper. For instance, in the current work, the authors assume a constant environment and thus a constant switching function. However, there is growing evidence of the critical role of a heterogeneous and dynamic environment, such as in relation to oxygen distribution and hypoxia or glucose and nutrients. Discussing on this point and exploring how the model could be adapted to incorporate environmental heterogeneity and how this might influence tumor dynamics would be insightful.

We agree that discussing perspectives on future research strengthens the paper and have consequently expanded the discussion:

- *L. 477ff: Future research can build on this foundation by exploring additional aspects of tumor evolution and therapy response. For instance, incorporating explicit dependencies on specific cell-microenvironment interactions and additional environmental factors, such as diffusing nutrients and oxygen, could provide a more comprehensive understanding of tumor growth's complex dynamics. This would lead to a more complex, nonlinear, non-monotonic, phenotypic switch function with multiple inputs, potentially resulting in even richer dynamics resulting from the dynamic and heterogeneous tumor microenvironment.*

However, we would like to point out that since the phenotypic switch depends on the local cell density, this work already captures aspects of the heterogeneous and dynamic tumor microenvironment. We do agree, though, that when applying our model to specific inputs of the microenvironment, such as the oxygen distribution, additional sources of heterogeneity would need to be considered, e.g., the spatial distribution of blood vessels.

In conclusion, while the paper is an interesting piece of research, addressing these suggestions will strengthen the paper's impact and relevance to the field. Therefore, I recommend the manuscript for publication after addressing these minor revisions.

Reviewer #3

This paper deals with an essential, and yet unsolved, problem in oncology, namely the effect of heterogeneity in the growth and response to therapy of tumours. This paper presents a relatively simple model of tumour growth. The simplifications introduced allow for a degree of analytical progress which helps to understand and interpret the simulation results. This paper is worthy contribution to the existing literature in the subject.

I have only one minor comment regarding the model formulation that needs some clarification. It refers to the hypothesis that local density is a proxy for the effect of the microenvironment. This assumption is a reasonable one and allows the author to gain an analytical handle on the model. However, the relation between cell behaviour and the microenvironment is a complex, non-linear one. It would be useful to include a discussion on the limitations of this asumption and how the model could be modified to account for more detailed mathematical descriptions of the cell-to-environment relationship.

We thank the reviewer for their positive evaluation. We agree that this aspect should've been discussed more, which was also remarked by the other reviewers. Therefore, we have added an additional subsection to the discussion. Please also refer to the reply to the other reviewers' remarks about this point.
